# COVID-19: spot urine rather than bronchoalveolar lavage fluid analysis?

**DOI:** 10.1186/s13054-021-03579-5

**Published:** 2021-04-30

**Authors:** Faeq Husain-Syed, Claudio Ronco, Thorsten Wiech, Matthias Hecker, Werner Seeger

**Affiliations:** 1 Department of Internal Medicine II, University Hospital Giessen and Marburg, Justus Liebig University Giessen, Klinikstrasse 33, 35392 Giessen, Germany; 2 International Renal Research Institute of Vicenza (IRRIV), Department of Nephrology, Dialysis and Transplantation, AULSS8 Regione Veneto, San Bortolo Hospital, Viale Rodolfi, 37, 36100 Vicenza, Italy; 3 Department of Medicine (DIMED), Università Di Padova, Via Giustiniani, 2, 35128 Padua, Italy; 4 Institute of Pathology, Nephropathology Section, University Hospital Hamburg Eppendorf, Martinistrasse 52, 20246 Hamburg, Germany; 5 Member of the German Center for Lung Research, Universities of Giessen and Marburg Lung Center, Klinikstrasse 33, 35392 Giessen, Germany; 6 Department of Lung Development and Remodeling, Max Planck Institute for Heart and Lung Research, Ludwigstrasse 43, 61231 Bad Nauheim, Germany

Severe acute respiratory syndrome coronavirus type-2 (SARS-CoV-2), which is responsible for coronavirus disease 2019 (COVID-19), has infected over 130 million people and caused more than 2.8 million deaths globally (as of April 4th 2021, according to WHO data). Identifying risk factors and prognostic markers for the development of COVID-19 disease and its sequelae is urgent in order to enable early identification and monitoring of high-risk patients.

While COVID-19 is most commonly characterized as a respiratory illness, extrapulmonary manifestations are a prominent part of its clinical spectrum. Kidney involvement is common in COVID-19. Hematuria, proteinuria, and acute kidney injury (AKI) are frequently reported in hospitalized COVID-19 patients, with incidence rates of 11.3–40.9%, 42.1–43.9%, and 5.1–36.6%, respectively, and they are associated with mortality [1]. The ACE2 receptor, which is the cellular entry point for SARS-CoV-2, is similarly expressed on bronchial and alveolar epithelial cells, as well as on podocytes and renal tubular epithelial and endothelial cells; therefore, direct viral tissue damage is a plausible mechanism of kidney injury (Fig. 1; [1–9]).

**Fig. 1:**
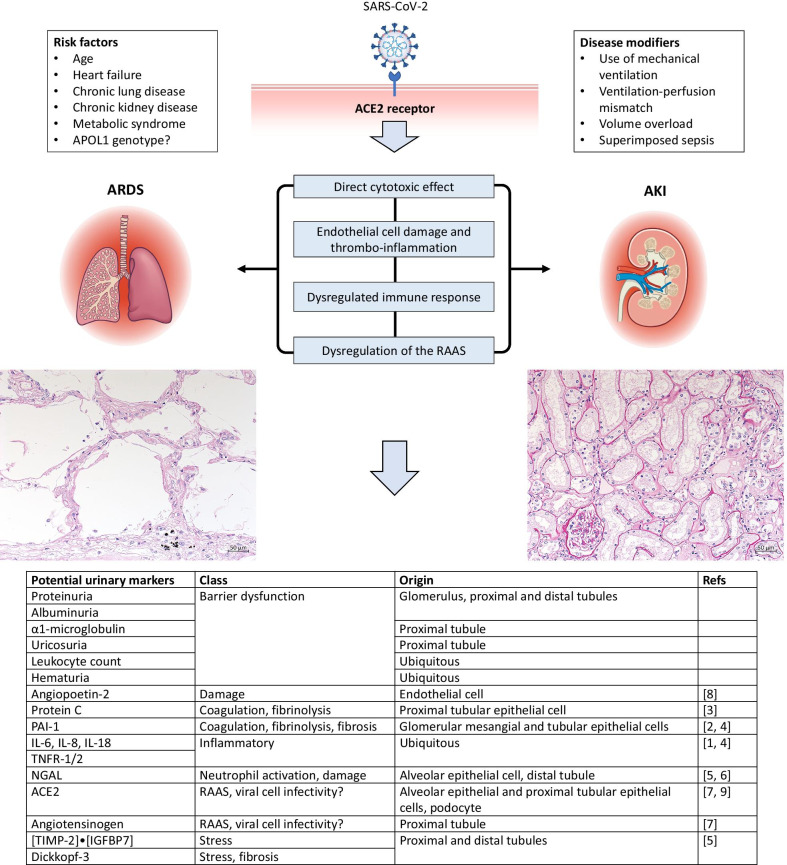
Pulmonary–renal damage patterns of COVID-19. Clinical and histopathological data of COVID-19-associated ARDS and AKI indicate common etiological factors, including direct virus-mediated cell damage, endothelial damage and thrombo-inflammation, dysregulation of the immune response and hyperinflammation, and dysregulation of the renin–angiotensin–aldosterone system. In addition, use of mechanical ventilation, hemodynamic instability, volume overload, superimposed sepsis, and collapsing glomerulopathy have all been implicated in glomerular disease and tubular damage in COVID-19. In contrast to bronchoalveolar lavage fluid, urine is accessible at any time point for non-invasive assessment of organ involvement. The urinary tubuloglomerular pattern may mirror the bronchoalveolar damage pattern and, thus, allow characterization and assessment of the stage of the pulmonary disease by means of serial urine analysis. The table summarizes potential candidate urinary markers that could inform lung injury in the setting of COVID-19-associated ARDS. However, it is unclear which marker is most accurate for this purpose and what cut-offs are needed, and this requires further investigation. The left histopathological image shows diffuse alveolar damage with hyaline membranes at the alveolar walls as morphologic correlate of ARDS. The right histopathological image shows acute tubular injury with flattened brush borders and cytoplasm of the epithelial cells in dilated tubules as morphologic correlate of AKI. *ACE2* angiotensin-converting enzyme 2, *AKI* acute kidney injury, *ARDS* acute respiratory distress syndrome, *COVID-19* coronavirus disease 2019, *IGFBP7* insulin-like growth factor-binding protein 7, *IL* interleukin, *PAI-1* plasminogen activator inhibitor-1, *NGAL* neutrophil gelatinase-associated lipocalin, *RAAS* renin–angiotensin–aldosterone system, *SARS-CoV-2* severe acute respiratory syndrome coronavirus type 2, *TIMP-2* tissue inhibitor of metalloproteinases-2, *TNFR* tumor necrosis factor receptor

The parallelism between the bronchoalveolar and tubuloglomerular processes in COVID-19 is not surprising, since these structures are ontogenetically closely related and simultaneous lung and kidney damage patterns have been observed in other clinical conditions. This phenomenon is referred to by the umbrella term “pulmonary–renal syndromes”, which summarizes diverse disease states associated with alveolar hemorrhage and glomerulonephritis. A known prototype of pulmonary–renal syndromes is Goodpasture disease, which is an autoimmune condition associated with the NC1 domain of the alpha-3 chain of type IV collagen, but the restricted tissue distribution of the alpha-3 chain generally limits injury to the glomeruli and alveoli. Autoimmunity has also been implicated in COVID-19-associated glomerular injury mediated by immunocomplexes of the viral antigen or virus-induced immunological effects, indicated by the development of collapsing glomerulopathy in subjects infected with SARS-CoV-2 who have high-risk variants of the *APOL1* genotype [1].

In contrast to the bronchoalveolar compartment, the tubuloglomerular compartment is accessible at any time for non-invasive diagnostics that involve urine analysis. There are two important questions concerning the use of urine analysis for COVID-19:

1. Can a differential urine analysis be used to predict COVID-19-associated AKI, and can it provide information on whether the kidney damage is caused by viral cytopathogenicity, by local/systemic immune reactions, or by alternative mechanisms?
2. Does the tubuloglomerular damage pattern mirror the bronchoalveolar damage pattern and, thus, allow characterization and assessment of the stage of the pulmonary disease process by means of serial urine analysis?

Prospective multicenter trials would be required to answer these questions. Specifically, analysis of pulmonary/renal functional parameters as well as biomarker expression by urine analyses would be required to inform lung injury and potentially use renal stress/damage patterns to support diagnosis. Serial urine analyses may include: (1) traditional markers of tubuloglomerular and vascular barrier dysfunction; (2) markers of coagulation/fibrinolysis; (3) cytokine profiles indicative of immunological damage mechanisms; (4) markers of renin–angiotensin–aldosterone system activation potentially reflecting viral cell infectivity; and (5) alveolar and tubular stress/damage biomarkers indicating acute and permanent structural tissue damage. These urine parameters would then be associated with pulmonary, renal, and systemic parameters and their course, as well as the features of biopsy (and autopsy) tissue samples of both organs. Finally, these biomarker profiles would require validation in subsequent prospective studies in terms of their sensitivity and specificity.

Surprisingly, although a few studies have reported the use of urinary biomarkers to prognosticate COVID-19-associated critical illness and AKI [1], there is a paucity of data examining their value to monitor the pattern of COVID-19-associated lung damage. Various ARDS biomarkers indicative of different pathways of injury have been identified, but none of these have been studied in urine samples from ARDS patients [8].

Disruption of the alveolar-capillary barrier is a pathological hallmark of ARDS that is associated with accumulation of protein-rich inflammatory edematous fluid in the alveolar space, and is associated with disease severity; this feature is also seen in COVID-19-associated ARDS [10]. Likewise, SARS-CoV-2 causes tubular barrier dysfunction [11], and features of proximal tubule dysfunction (e.g., proteinuria, α1-microglobulin excretion, uricosuria) are independently associated with risk of respiratory failure and AKI [11–13]. In a small study on COVID-19 patients, proteinuria was found to be significantly correlated with the driving pressure, indicating its potential role as a surrogate marker of lung strain in ARDS [13]. However, it is not clear whether the degree of proteinuria is correlated with the total protein concentration of bronchoalveolar lavage.

It has long been known that high tidal volume ventilation in classical ARDS is associated with the release of various mediators, which may play a role in mediating AKI [4]. There is also experimental evidence that injurious ventilatory strategies can induce alveolar damage and renal epithelial cell apoptosis and dysregulation of Fas ligands [14]. Furthermore, a correlation between changes in levels of soluble Fas ligand and serum creatinine can be observed in patients with classical ARDS. Thus, some data point to the existence of a link between respiratory failure, ventilatory strategies, biotrauma, circulating mediators, and AKI. The topic that needs to be investigated in the future is whether there is a link between urinary biomarkers and ARDS severity in COVID-19 beyond the effects of mechanical ventilation on the kidney.

Finally, detection of SARS-CoV-2 mRNA in post-mortem specimens is associated with shorter survival time and increased incidence of AKI, indicating renal tropism of SARS-CoV-2 [1]. One implication of this finding is that urine testing for viral mRNA might help in the risk stratification of COVID-19 patients, but current data indicate that viral shedding in urine is rare [15].

Analysis of urine samples is straightforward, and the samples are easy to obtain. Importantly, minimizing the use of bronchoalveolar lavage fluid analysis may reduce the risk of transmission of SARS-CoV-2 infection through aerosol generation during bronchoscopy. Future research should aim to investigate whether the pathogenicity pattern of the tubuloglomerular compartment as indicated by urine analysis corresponds to that of the bronchoalveolar compartment, and whether such urinalysis is suitable for serial assessment of SARS-CoV-2 disease severity.

## Data Availability

Not applicable.

## References

[1] Nadim MK, Forni LG, Mehta RL, Connor MJ, Liu KD, Ostermann M, Rimmele T, Zarbock A, Bell S, Bihorac A (2020). COVID-19-associated acute kidney injury: consensus report of the 25th Acute Disease Quality Initiative (ADQI) Workgroup. Nat Rev Nephrol.

[2] Ha H, Oh EY, Lee HB (2009). The role of plasminogen activator inhibitor 1 in renal and cardiovascular diseases. Nat Rev Nephrol.

[3] Radtke KP, Fernandez JA, Greengard JS, Tang WW, Wilson CB, Loskutoff DJ, Scharrer I, Griffin JH (1994). Protein C inhibitor is expressed in tubular cells of human kidney. J Clin Investig.

[4] Liu KD, Glidden DV, Eisner MD, Parsons PE, Ware LB, Wheeler A, Korpak A, Thompson BT, Chertow GM, Matthay MA (2007). Predictive and pathogenetic value of plasma biomarkers for acute kidney injury in patients with acute lung injury. Crit Care Med.

[5] Ostermann M, Zarbock A, Goldstein S, Kashani K, Macedo E, Murugan R, Bell M, Forni L, Guzzi L, Joannidis M (2020). Recommendations on acute kidney injury biomarkers from the acute disease quality initiative consensus conference: a consensus statement. JAMA Netw Open.

[6] Xiao R, Chen R (2017). Neutrophil gelatinase-associated lipocalin as a potential novel biomarker for ventilator-associated lung injury. Mol Med Rep.

[7] Kobori H, Nangaku M, Navar LG, Nishiyama A (2007). The intrarenal renin-angiotensin system: from physiology to the pathobiology of hypertension and kidney disease. Pharmacol Rev.

[8] Spadaro S, Park M, Turrini C, Tunstall T, Thwaites R, Mauri T, Ragazzi R, Ruggeri P, Hansel TT, Caramori G (2019). Biomarkers for acute respiratory distress syndrome and prospects for personalised medicine. J Inflamm (Lond).

[9] Reindl-Schwaighofer R, Hodlmoser S, Eskandary F, Poglitsch M, Bonderman D, Strassl R, Aberle JH, Oberbauer R, Zoufaly A, Hecking M (2021). Angiotensin-converting enzyme 2 (ACE2) elevation in severe COVID-19. Am J Respir Crit Care Med.

[10] Borczuk AC, Salvatore SP, Seshan SV, Patel SS, Bussel JB, Mostyka M, Elsoukkary S, He B, Del Vecchio C, Fortarezza F (2020). COVID-19 pulmonary pathology: a multi-institutional autopsy cohort from Italy and New York City. Mod Pathol.

[11] Werion A, Belkhir L, Perrot M, Schmit G, Aydin S, Chen Z, Penaloza A, De Greef J, Yildiz H, Pothen L (2020). SARS-CoV-2 causes a specific dysfunction of the kidney proximal tubule. Kidney Int.

[12] Pei G, Zhang Z, Peng J, Liu L, Zhang C, Yu C, Ma Z, Huang Y, Liu W, Yao Y (2020). Renal involvement and early prognosis in patients with COVID-19 pneumonia. J Am Soc Nephrol.

[13] Husain-Syed F, Wilhelm J, Kassoumeh S, Birk HW, Herold S, Vadasz I, Walmrath HD, Kellum JA, Ronco C, Seeger W (2020). Acute kidney injury and urinary biomarkers in hospitalized patients with coronavirus disease-2019. Nephrol Dial Transplant.

[14] Imai Y, Parodo J, Kajikawa O, de Perrot M, Fischer S, Edwards V, Cutz E, Liu M, Keshavjee S, Martin TR (2003). Injurious mechanical ventilation and end-organ epithelial cell apoptosis and organ dysfunction in an experimental model of acute respiratory distress syndrome. JAMA.

[15] Trypsteen W, Van Cleemput J, Snippenberg WV, Gerlo S, Vandekerckhove L (2020). On the whereabouts of SARS-CoV-2 in the human body: a systematic review. PLoS Pathog.

